# Illuminating New Frontiers: Exploring the Photosensitizing Potential of *Passiflora* Species in Combating Methicillin‐Resistant *Staphylococcus aureus* (MRSA) and Their Infection in Senescent Mice

**DOI:** 10.1002/adbi.202500254

**Published:** 2025-08-29

**Authors:** Caroline Vieira Gonçalves, Maria Poliana Leite Galantini, Igor Pereira Ribeiro Muniz, Paulo Henrique Bispo Lima, Israel Souza Ribeiro, Maria Eduarda Santos de Oliveira, Caio Oliveira Lopes de Magalhães, Maria Elisa Santos Flores, Samara Lopes de Oliveira, Catarina Silva Guimarães, Paulinne Moreira Lima, Luísa Carregosa Santos, Daiana Silva Lopes, Juliano Geraldo Amaral, Robson Amaro Augusto da Silva

**Affiliations:** ^1^ Multidisciplinary Institute of Health Anísio Teixeira *Campus* ‐ Federal University of Bahia Rio de Contas Street, 58, Candeias Vitória da Conquista Bahia CEP: 45.029‐094 Brazil; ^2^ Paulo Freire *Campus* ‐ Federal University of Southern Bahia Joana Angélica Square, São José, 250 Teixeira de Freitas Bahia CEP: 45.988‐058 Brazil

**Keywords:** antimicrobial photodynamic therapy, methicillin‐resistant *Staphylococcus aureus*, *Passiflora cincinnata*, senescence

## Abstract

Antimicrobial Photodynamic Therapy (aPDT) has become a potential alternative for treating multidrug‐resistant bacterial skin infections, such as those caused by methicillin‐resistant *Staphylococcus aureus* (MRSA), which are at high risk in aging individuals. One of the main components of aPDT is an agent known as a photosensitizer (PS). Some plants with high flavonoid content are reported as PS. In the genus *Passiflora*, flavonoids are predominant, but their photosensitizing activity has yet to be described. This study investigates the photosensitizing potential of extracts from *Passiflora edulis*, *Passiflora alata*, and *Passiflora cincinnata*. The butanolic fraction of *P. cincinnata* undergoes in vivo evaluation against intradermal MRSA infection in a senescent murine model (C57BL/6). In vitro assays determine the photoactivatable concentrations and their cytotoxicity. In vivo, MRSA‐infected mice are divided into control, *P. cincinnata*‐treated, and aPDT‐treated groups. Subsequent assessments include cytokine levels, bacterial load, and cellular infiltrate in the ear. The *P. cincinnata*‐treated group exhibits improved bacterial control, reduced leukocyte infiltration, and less weight loss. The aPDT group demonstrates a unique cytokine correlation profile, featuring more negative correlations among pro‐inflammatory cytokines and interleukin‐10. *P. cincinnata* emerges as an effective photosensitizer for aPDT in a senescent model and highlights the potential of underexplored plant‐derived photosensitizers.

## Graphical abstract

This study investigates the photosensitizing potential of *Passiflora cincinnata* in Antimicrobial Photodynamic Therapy (aPDT) against skin infection caused by Methicillin‐resistant *Staphylococcus aureus* (MRSA) in a senescent murine model. The *P. cincinnata* exhibits antimicrobial activity, with reduced bacterial load, lower leukocyte infiltration, and less weight loss, highlighting its therapeutic potential in resistant infections.

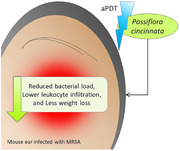

## 1 Introduction

The elderly represent the population with the highest risk of infections and the worst prognosis for infectious diseases.^[^ ^1^ ^]^ This includes the high risk of skin and soft tissue infections due to several factors, with multiple comorbidities and skin conditions predisposing them to develop these infections, which are usually caused by *S aureus*.^[^ ^2^, ^3^ ^]^ An example of these factors is skin thickness, which changes with age, tending to become thinner over time.^[^ ^4^ ^]^ Furthermore, aging reduces the functional capacity of the skin, resulting in the attenuation of its protective effects and immunological dysfunction. Consequently, opportunistic skin infections and the development of chronic wounds, which serve as entry points for infectious agents, are more common in elderly patients.^[^ ^5^ ^]^

*S. aureus* is a pathogen that causes skin and soft tissue infections as well as serious invasive diseases such as sepsis and endocarditis.^[^ ^6^ ^]^ Despite being one of the first microorganisms to be controlled with the discovery of antibiotics, it has become one of the most important species in the context of hospital and community infections due to its ability to adapt and resist, as is the case with methicillin‐resistant *Staphylococcus aureus* (MRSA).^[^ ^7^, ^8^ ^]^ In parallel with the difficulty in finding effective drugs against MRSA, there is significant concern about infection control in immunosenescent individuals.^[^ ^9^, ^10^ ^]^

In this regard, the World Health Organization (WHO) has identified MRSA as a high priority for the development of new therapeutics.^[^ ^11^ ^]^ New antimicrobials emerged after the discovery of sulfonamides and penicillin in the early 20th century, marking the beginning of the golden age of new‐class conquest, which peaked in the mid‐1950s.^[^ ^12^, ^13^ ^]^ However, since then, there has been a gradual decline in the discovery and development of new antibiotics, which have not kept pace with the emergence of new resistant strains.^[^ ^14^ ^]^ This factor contributes to its indiscriminate use, culminating in the current antimicrobial resistance crisis.^[^ ^15^ ^]^ Furthermore, most small molecules currently in clinical development or recently approved are modifications of existing chemical structures.^[^ ^16^ ^]^ In the face of this growing threat, researchers have explored various innovative alternatives to combat pathogens such as MRSA. These efforts range from the engineering of materials with novel antibacterial functionalities to the investigation of alternative therapies,^[^ ^17^, ^18^, ^19^ ^]^ including compounds of natural origin.^[^ ^20^, ^21^, ^22^ ^]^

In light of these issues, Antimicrobial Photodynamic Therapy (aPDT) has emerged as a potential alternative for treating multidrug‐resistant bacterial infections.^[^ ^23^ ^]^ In the skin, aPDT has been shown to reduce MRSA colonies.^[^ ^24^, ^25^, ^26^, ^27^, ^28^ ^]^ However, no study has aimed at this analysis in a murine model of senescence with intradermal infection. The development of this model is important since the murine model of MRSA infection in senescent mice captures the characteristic clinical finding that elderly individuals are more susceptible to invasive disease after cutaneous infection, which may result from grossly permeable barrier function and decreased innate immunity in these animals.^[^ ^29^ ^]^ Moreover, when combined with aPDT, it could provide necessary guidance for understanding therapies aimed at immunosenescence.^[^ ^30^, ^31^, ^32^ ^]^

aPDT employs a combination of photosensitizers with a specific light source, capable of generating molecules that act selectively.^[^ ^33^ ^]^ Therefore, one of the main components of PDT is the chemical agent called photosensitizer (PS).^[^ ^34^ ^]^ For it to be effective, the choice of photosensitizers is crucial. In this regard, studying new compounds is of fundamental importance for identifying new candidates for photosensitizers that may be suitable for controlling infections caused by antibiotic‐resistant bacteria.^[^ ^35^, ^36^, ^37^ ^]^ Recent studies have demonstrated the ability of certain compounds, such as plants with a high flavonoid content, to exhibit antimicrobial photodynamic activity.^[^ ^24^ ^]^

Phytochemical studies of *Passiflora* have demonstrated the presence of several chemical compounds such as alkaloids, saponins, and cyanogenic acids, the most frequently reported being: Flavonoids *C*‐glycosides.^[^ ^38^, ^39^ ^]^ This is the case of some *Passiflora* species, such as *P. edulis*, *P. alata*, and *P. cincinnata*, with no current description regarding their antimicrobial activity and photosensitizing capacity.^[^ ^40^ ^]^ Among these species, *Passiflora cincinnata* is the least reported in the literature, but it contains flavonoids in its major compounds.^[^ ^41^ ^]^ This is a native species of the Caatinga (a biome exclusive to Brazil) with a wide geographic distribution, and little is known about its bioactive compounds.^[^ ^42^ ^]^

Given the above, this study aimed to evaluate the photosensitizing activity of *P. edulis*, *P. alata*, and *P. cincinnata* extracts. In this way, we used *P. cincinnata* to assess its use in aPDT against an intradermal infection caused by MRSA in a murine model of senescence using the C57BL/6 lineage.

## 2 Results

### 2.1 The Optimal Absorption of *Passiflora spp*. in the Visible Light Spectrum Was Observed in the Blue Range

**Figure 1:**
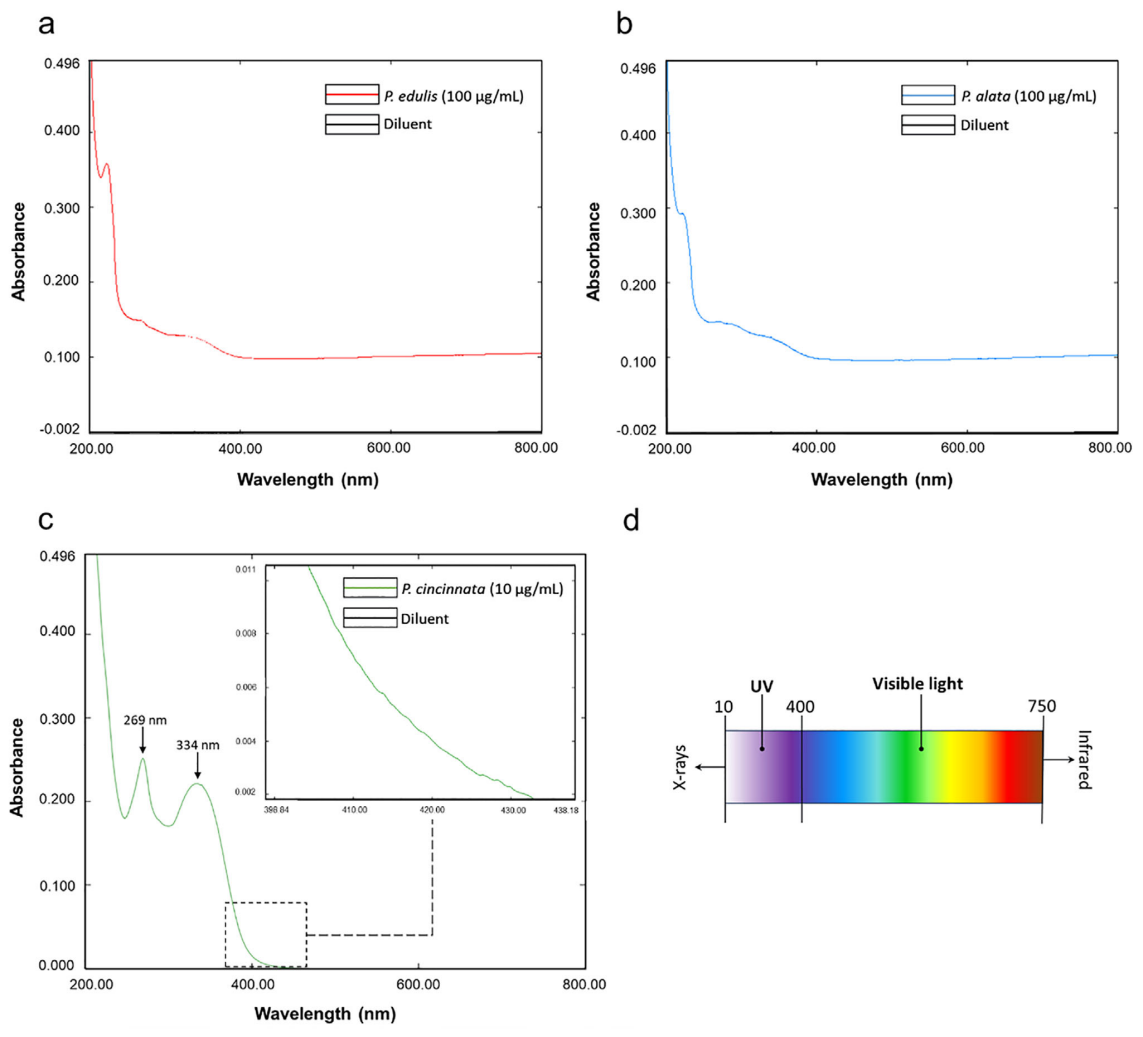
Scanning of *Passiflora* extracts in the UV–vis spectrum, evaluating the interval between 200 and 800 nm. Blue line: *Passiflora* extract. Black line: Diluent (distilled water). a) *P. edulis* scan. b) *P. alata* scan. c) *P. cincinnata* scan. d) Wavelength ranges of electromagnetic radiation.

The scanning analysis was performed using a UV–vis absorption spectrophotometer to estimate at which wavelengths the *Passiflora* extracts absorb light, evaluating the interval between 200 and 800 nm. All the species of *Passiflora* tested in the study showed a growing peak in the blue light range, from 400 nm. However, *P. edulis* (**Figure** **1**a) and *P. alata* (Figure 1b) demonstrated a lower absorption in this region compared to *P. cincinnata* (Figure 1c), necessitating their evaluation at different concentrations (concentrations were 100 µg mL^−1^ for *P. edulis* and *P. alata*, and 10 µg mL^−1^ for *P. cincinnata*). This was due to saturation observed in the *P. cincinnata* graph when tested at 100 µg mL^−1^. For *P. cincinnata*, the highest light absorption peak was observed in the UVA range at 269.50 and 334 nm. Although an increase at 400 nm corresponds to blue light, the most prominent peaks are observed in the UVA range. This does not exclude the possibility of photosensitizing activity within the visible light spectrum (Figure 1d).

### 2.2 Non‐Cytotoxic Effects of *Passiflora* Extracts on HUVEC Cells In Vitro

**Figure 2:**
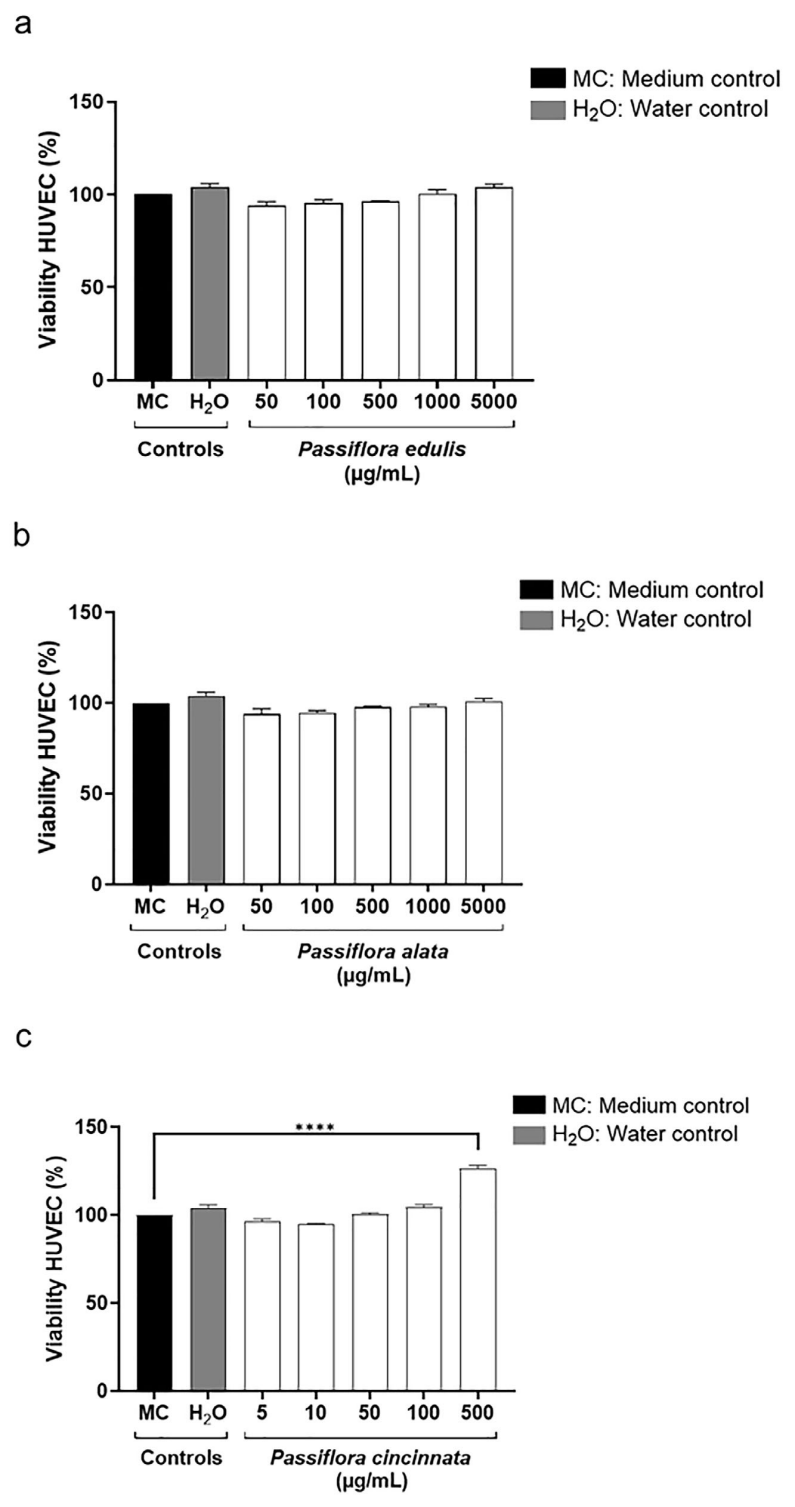
Cytotoxicity evaluation of *Passiflora* extracts in HUVEC cell culture by MTT assay. The HUVEC cells were seeded at 3 × 10^4^ per well in 96‐well microplates. After treatment, cells were incubated with MTT to assess the viability of the HUVEC cells. a) The *P. edulis* extract. b) The *P. alata* extract. c) The butanolic fraction of *P. cincinnata* extract.

To enable the use of *Passiflora* extracts in vivo, we conducted cytotoxicity tests in HUVEC cell cultures, employing different concentration ranges. HUVEC cells were specifically chosen because they are nucleated, making them a robust model for assessing potential cytotoxic effects. No cytotoxicity was observed for *P. edulis* (**Figure** **2**a), *P. alata* (Figure 2b), *P. cincinnata* (Figure 2c), or their diluents.

### 2.3 *Passiflora* Extracts Tested Have a Photosensitizing Capacity Against MRSA

**Figure 3:**
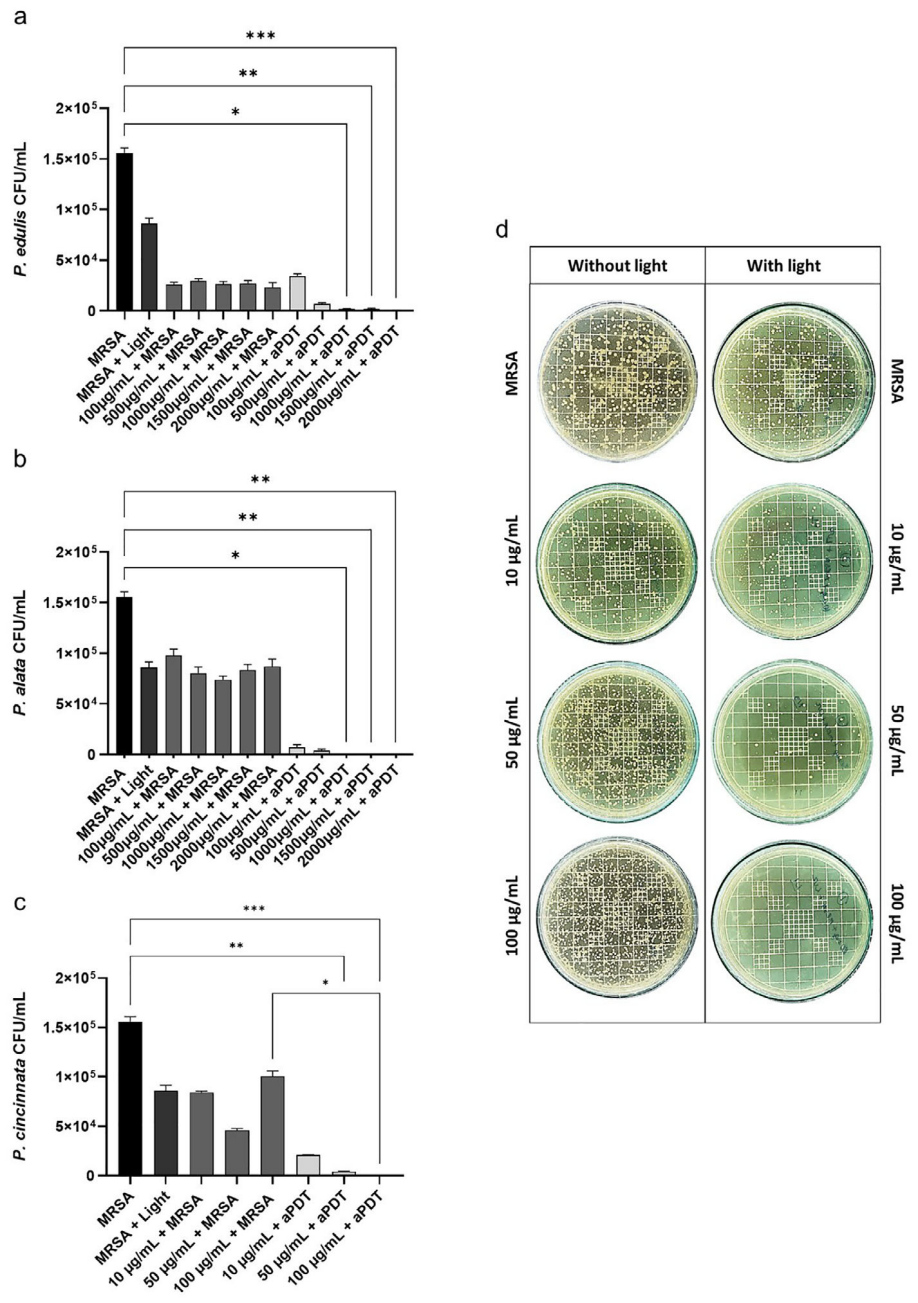
In vitro experiment evaluating the photosensitizing activity of *Passiflora* extracts. The *Passiflora* extracts tested were diluted in distilled water and used in concentrations of 2000, 1500, 1000, 500, and 100 µg mL^−1^ for *P. edulis* and *P. alata*. In contrast, they were tested at 100, 50, and 10 µg mL^−1^ for *P. cincinnata*. a) The photosensitizing activity of *P. edulis*. b) The photosensitizing activity of *P. alata*. c) Photosensitizing activity of the butanolic fraction of *P. cincinnata*. d) Culture plate of photosensitizing activity of butanolic fraction of *P. cincinnata*. The *p*‐value was considered significant when represented by asterisks, where ^*^ *p* < 0.05; ^**^ *p* < 0.01; ^***^ *p* < 0.001.

The results of the assays indicate that various *Passiflora* species exhibit photosensitizing activity against Methicillin‐resistant *Staphylococcus aureus* (MRSA). This activity was notably enhanced when the extracts were exposed to blue light, particularly at lower concentrations. Specifically: *P. edulis* (**Figure** **3**a) and *P. alata* (Figure 3b) demonstrated a more effective photosensitizing capacity at a concentration of 1000 µg/mL. *P. cincinnata* (Figure 3c,d) exhibited significant photosensitizing activity at a concentration of 100 µg mL^−1^. These findings indicate that different *Passiflora* species vary in their photosensitizing effectiveness, with higher concentrations of *P. edulis* and *P. alata* required to achieve comparable effects to those observed with lower concentrations of *P. cincinnata*.

### 2.4 Animals with *P. cincinnata* Had Less Weight Variation

**Figure 4:**
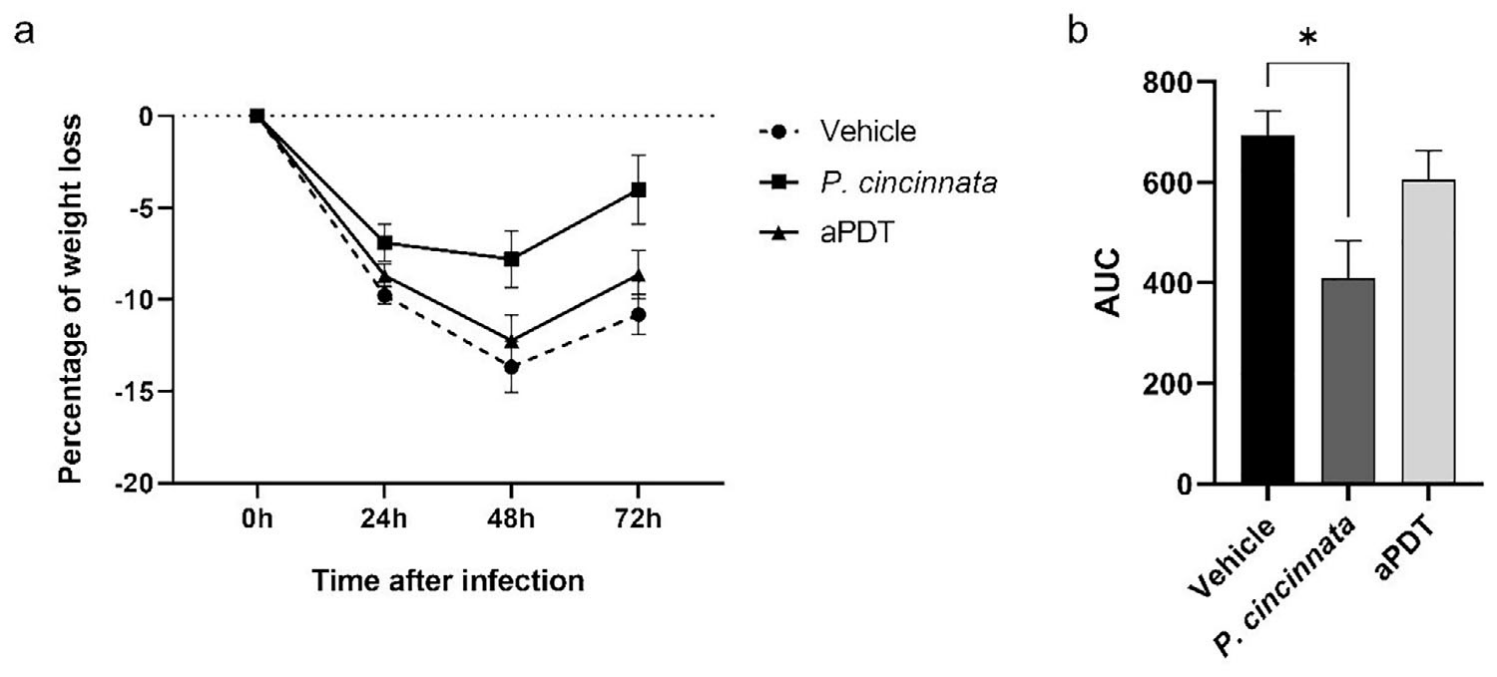
Animal weight after intradermal infection and treatment. a) Weight loss percentage of each animal, considering its initial weight. b) The area under the percentage curve (AUC) for weight loss in each animal, referring to graph A. The *p*‐value was considered significant when represented by asterisks, where ^*^ *p* < 0.05.

The results detail the evaluation of body weight over time and the Area Under the Curve (AUC) for weight loss in the treatment groups with Vehicle, *P. cincinnata*, and aPDT. Analysis of the percentage of weight loss reveals that the Vehicle group demonstrated the most significant loss, peaking at 48 h post‐infection, with a subsequent but limited recovery (**Figure** **4**a). In contrast, the *P. cincinnata*‐treated groups exhibited the least weight loss, also peaking at 48 h, followed by a more pronounced and robust recovery by 72 h. These findings are corroborated by the AUC for weight loss, an integrated indicator of the magnitude and duration of weight loss, where lower values denote less total loss. The AUC data reinforce that treatment with *P. cincinnata* was the most effective in mitigating infection‐induced weight loss, as evidenced by both the lower percentage of weight loss over time and the smaller AUC, with statistical significance relative to the Vehicle group (Figure 4b).

### 2.5 Control Animals Had a Higher Leukocyte Cell Infiltrate and a Higher Bacterial Load

**Figure 5:**
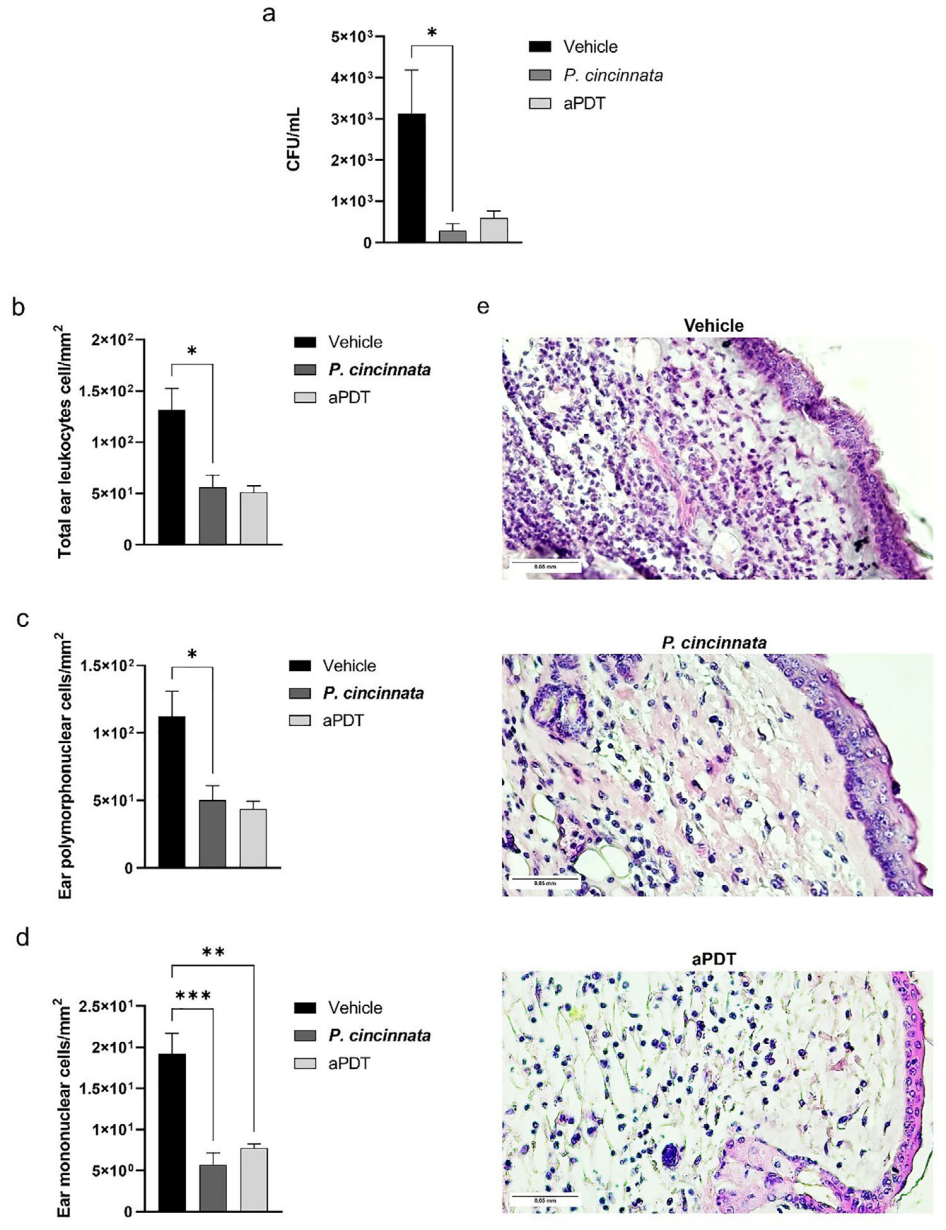
Bacterial load and leukocyte cell influx. Ears were sectioned to 4 µm, and sections were stained with hematoxylin and eosin. The stained sections were viewed under optical microscopy using a 40X objective. a) Bacterial load of the ear macerate. b) Total count of all leukocyte cells present in the infiltrate. c) The number of polymorphonuclear leukocytes. d) The number of mononuclear leukocytes. e) Representative photomicrographs of the infiltrates taken with the 40X objective representing the vehicle, *P. cincinnata*, and aPDT groups. The *p*‐value was considered significant when represented by asterisks, where ^*^ *p* < 0.05; ^**^ *p* < 0.01; ^***^ *p* < 0.001.

Animals in the control (Vehicle) group showed a higher bacterial load (**Figure** **5**a) and a greater number of total leukocyte infiltrates (Figure 5b) and polymorphonuclear leukocytes (Figure 5c) compared to the group treated with *P. cincinnata*. There was also an increase in the number of mononuclear cells (Figure 5d) in the control group compared to animals treated with *P. cincinnata* and aPDT. In all results, the Vehicle group exhibited a significantly higher count compared to the treatments with the purified butanolic fraction of *P. cincinnata* and aPDT, indicating a reduction in inflammatory cell infiltration in treated animals. Histological images (Figure 5d) show tissue morphology and the presence of inflammatory cells, corroborating the quantitative results.

### 2.6 Cytokine Interacted Differently among Them in Draining Lymph Nodes

**Figure 6:**
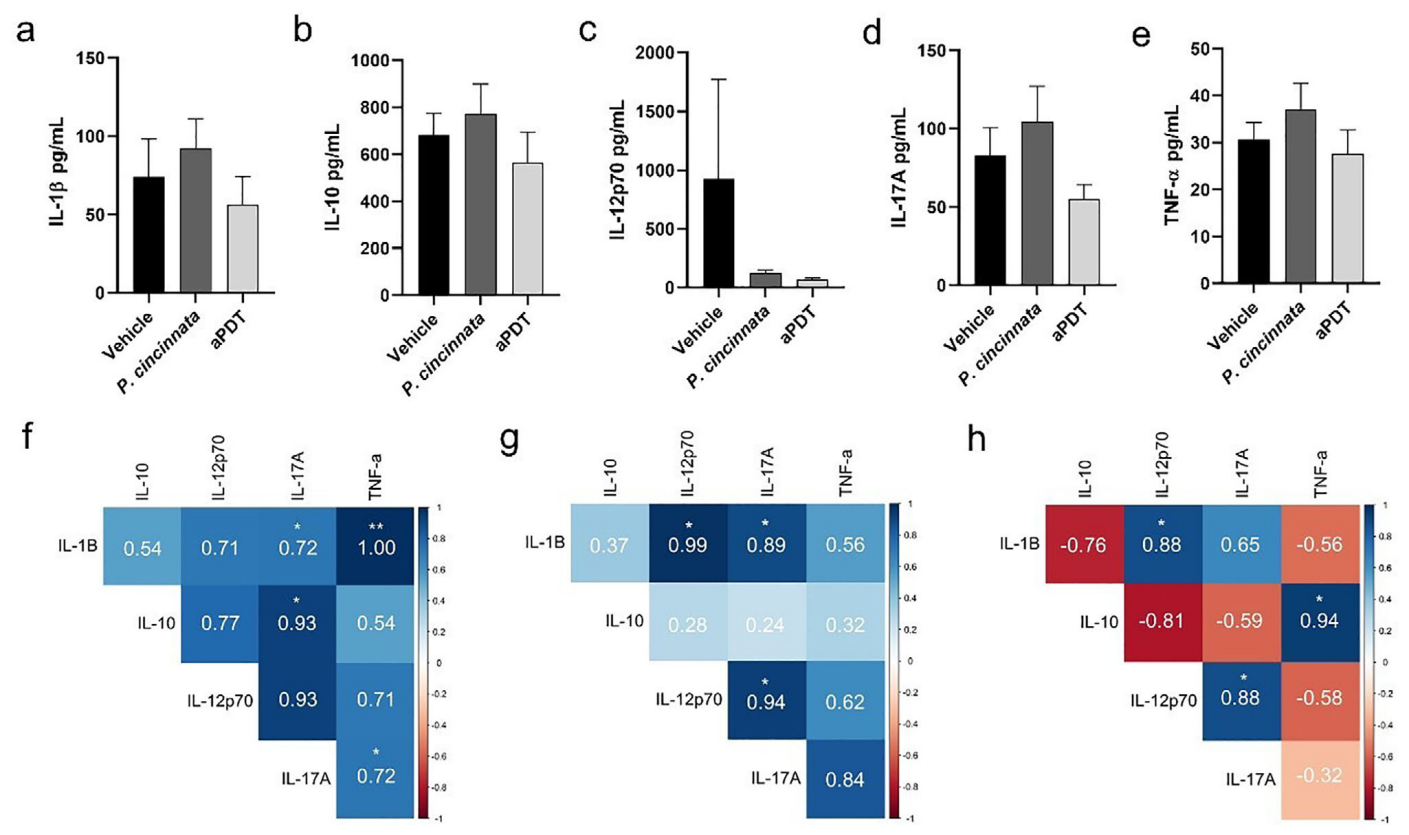
Cytokine assays in the ear‐draining lymph nodes. Lymph nodes were macerated in 1 mL of sterile saline and centrifuged. a) Lymph node supernatant samples were used to measure cytokines (TNF‐α, IL‐1β, IL‐12p70, IL‐17A, IL‐10) by ELISA. TNF‐α. b) IL‐1β. c) IL‐12p70. d) IL‐17A. e) IL‐10. f) Cytokine correlations in the Vehicle group. g) *P. cincinnnata* group cytokine correlations. h) Cytokine correlations in the aPDT group. Corrplot version 0.84 was used for the correlation. Positive correlations were indicated in blue, and negative correlations in red. Darker color tones indicate higher r values. The *p*‐values (p) were indicated based on Spearman or Pearson correlation tests (^*^ *p* < 0.05; ^**^ *p* < 0.01).

We observed that the animals did not have differences in production for all cytokines analyzed: IL‐1β (**Figure** **6**a), IL‐12p70 (Figure 6b), IL‐17A (Figure 6c), IL‐10 (Figure 6d), and TNF‐α (Figure 6e). In contrast, we observed differences in cytokine correlations among the vehicle (Figure 6f), *P. cincinnata* (Figure 6g), and aPDT (Figure 6h) groups. The positive correlations of IL‐12p70 with IL‐17 and IL‐12p70 with IL‐1β are present only in the *P. cincinnata* (*r* = 0.94 and *r* = 0.99) and aPDT (*r* = 0.88 and *r* = 0.88) groups, and are absent in the Vehicle group. We also observe the positive correlation of IL‐10 with IL‐17A in the Vehicle group (*r* = 0.93) and TNF‐α with IL‐1β (*r* = 1.00). The IL‐17A with IL‐1β correlates in both the Vehicle (*r* = 0.72) and *P. cincinnata* (*r* = 0.89) groups. In general, the movement of correlations differs between groups; there are more negative correlations in the aPDT group.

## 3 Discussion

The results of this unprecedented study reveal the photosensitizing potential of different *Passiflora* species (*P. edulis*, *P. alata*, and *P. cincinnata*) against MRSA. Among the species analyzed, *P. cincinnata* (and its butanolic fraction) was selected for an in‐depth investigation of its efficacy as a photosensitizer against MRSA infections in a murine senescence model. To our knowledge, this research is pioneering in exploring the photosensitizing properties of these species and the application of aPDT in a senescence model, particularly in animals (C57BL/6) with high melanin concentration.

In response to the challenge of antimicrobial resistance, researchers have explored various alternatives, seeking innovative solutions to combat pathogens such as *S. aureus*. This includes engineering of materials with novel antibacterial functionalities to the investigation of alternative therapies.^[^ ^17^, ^18^, ^19^ ^]^ Other studies have been dedicated to exploring natural extracts against MRSA, both in vitro and in vivo models. They demonstrate the potential of bioactives in expanding the arsenal of compounds that may be promising against resistant microorganisms. The constituents of the extracts can act synergistically, enhancing the photosensitizing power.^[^ ^24^, ^43^, ^44^ ^]^ Thus, increasing its action at low concentrations as observed by Ribeiro et al. (2023). In this study, we selected a plant genus that has been underexplored in terms of its activity against resistant microorganisms, specifically the *Passiflora* genus. This highlights its substantial potential for further exploration in more detailed research.

*Passiflora* is a genus belonging to the Passifloraceae family, with many species widely used in folk medicine and several pharmacological activities described in the scientific literature. Some biological properties of *Passiflora* have been demonstrated through in vitro and in vivo studies, related to activities such as antioxidant, analgesic, antidepressant, sedative, anxiolytic, anti‐inflammatory, antimicrobial, antihypertensive, hepatoprotective, and antidiabetic effects.^[^ ^45^, ^46^ ^]^ Studies have identified several compounds, mainly the flavonoid C‐glycosides, responsible for these activities.^[^ ^47^ ^]^ From a search in the literature, it has been shown that species of the genus *Passiflora* have a high content of flavonoids.^[^ ^48^, ^49^ ^]^ Dos Santos et al. (2019) address that, possibly, extracts formed mainly through flavonoids may be responsible for the photodynamic activity of *Myrciaria clauriflora*.

However, no studies have explored the use of *Passiflora* species as photosensitizers. In Brazil, the species most commonly used in folk medicine are *P. alata* and *P. edulis*.^[^ ^50^ ^]^ Therefore, few studies investigate the possible biological effects of *P. cincinnata* extracts.^[^ ^51^ ^]^ Some of these demonstrate that extracts from the leaves, bark, and seeds of *P. cincinnata* have in vitro antimicrobial and anti‐inflammatory activities, which are attributed to their phenolic compounds.^[^ ^42^, ^52^ ^]^ More specifically, the in vitro study by Siebra et al. (2018) assessed the antibiotic‐modifying activity against *S. aureus* strains using a subinhibitory concentration of the extract. The extracts did not exhibit clinically relevant antimicrobial activity, with the minimum inhibitory concentration (MIC) being equal to or greater than 1024 µg mL^−1^.

Along the same lines, Siebra et al. (2014) showed that the antimicrobial activity of hydroalcoholic extracts of *P. cincinnata*, (from leaves, stems, bark, pulp, and seeds) did not reach clinical relevance.^[^ ^53^ ^]^ However, the hydroalcoholic extract of *P. cincinnata* was presented as an antibacterial agent when associated with antibiotics. Its action profile was altered by the decrease in the minimum inhibitory concentration (MIC) of conventional antibiotics, suggesting that it can be developed as a new therapeutic weapon, potentiating aminoglycosides and beta‐lactams, which are not very effective in the treatment of infections caused by MRSA.^[^ ^47^, ^52^ ^]^ In contrast, our in vitro results show that the butanolic fraction of photoactivated *P. cincinnata* at a concentration of 100 µg mL^−1^ significantly reduces the bacterial load. This indicates that photoactivation enhances its effect.

This also applies to *P. edulis* and *P. alata*, though at concentrations ten times higher than those observed in photoactivated *P. cincinnata*, which may be attributed to the bioactive compounds present in the latter species. A doctoral thesis sought to enhance the understanding of *P. cincinnata* chemistry through a phytochemical investigation. The study isolated and identified key compounds, including isoorientin, isovitexin, isoscoparin, isovitexin‐2″‐*O*‐β‐glucopyranoside, isovitexin‐2″‐*O*‐β‐xylopyranoside, and isoorientin‐2‐*O*‐β‐xylopyranoside, which may contribute to its observed effects. Additionally, the phytochemical profile of the leaves and stems was analyzed using HPLC‐DAD‐IES‐ITEMn, leading to the identification of 19 additional metabolites, primarily flavonoids and chlorogenic acid derivatives.^[^ ^54^ ^]^

Before testing the photosensitising activity, knowing which spectrum we could work with was necessary. By analyzing the best light absorption spectrum of *Passiflora* spp. tested, we found that the blue light spectrum is the closest to visible light in which there was light absorption, and for this reason, it was chosen to be analyzed in this study. The *P. cincinnata* showed a higher increasing absorption peak in this range than *P. edulis* and *P. alata*, demonstrating its great relevance in using aPDT. Therefore, research aimed at developing or using new photosensitizers becomes fundamental since the more photoactive compounds, the greater the possibilities for combating the infection caused by MRSA.^[^ ^24^, ^27^ ^]^ Thus, we found that among the species of the genus *Passiflora* studied, *P. cincinnata* stood out for having photosensitizing activity at lower concentrations than *P. edulis* and *P. alata* against MRSA. In addition, it was demonstrated that none of the *Passiflora* spp. tested were cytotoxic to eukaryotic cells.

Thus, *P. cincinnata* was chosen to be used in the infectious treatment of MRSA in senescent animals of the C57BL/6 lineage. Besides having immunosenescence, animals from this lineage could provide more knowledge about the possibilities of applying light‐based therapies in individuals with large amounts of melanin, as observed in the study developed by Muniz et al. (2021), which used the same strain in a type 1 diabetes *mellitus* model of intradermal infection and treated with PDT. The importance of ex vivo activation studies becomes evident, particularly in understanding the impact of melanin on PDT, as limited studies have examined this interaction.^[^ ^55^ ^]^ MRSA infection associated with senescence is challenging to control since it is related to the reduced effectiveness of the immune system in combating an infectious process.^[^ ^56^ ^]^ In assessing the bioactivity of *P. cincinnata* extracts and their application in aPDT with senescent animals, our findings indicate that treatment with *P. cincinnata* resulted in improved control of bacterial load, reduced leukocyte cell infiltration, and less weight loss throughout the MRSA infection process. Similarly, animals treated with aPDT exhibited trends comparable to those treated with *P. cincinnata* alone.

Aging individuals have several risk factors for skin and soft tissue infections. Changes in skin consistency and immunosenescence predispose this population to these infections. Changes in the dermal matrix, atrophy, senescence of dermal cells such as fibroblasts, and decreased synthesis coupled with accelerated breakdown of dermal collagen fibers increase susceptibility to skin infections. In addition, they have a high frequency of conditions associated with skin fragility, such as edema and trauma.^[^ ^1^, ^57^ ^]^ How advanced age contributes to invasive staphylococcal disease is not yet fully understood. Although many immunological defects (e.g., phagocytosis, oxygen reactive species) are associated with aging, the fundamental age‐dependent cellular changes responsible for these defects are unknown. However, it is well known that cells from elderly individuals have altered mitochondrial functions, which may affect their ability to respond to external stimuli, including pathogens.^[^ ^29^ ^]^

Despite these facts, we observed that the animals treated with *P. cincinnata* and aPDT had less weight variation, probably because they controlled the infectious process better, more quickly and effectively,^[^ ^58^ ^]^ not needing to recruit as many defense cells after 72 h of the infectious. These data are interesting since other studies observe a more significant infiltration of leukocyte cells, mainly polymorphonuclear cells, in the groups subjected to aPDT, after 72 h of infection. However, it is worth mentioning that it was in a diabetes model.^[^ ^25^ ^]^ The *P. cincinnata* can probably eliminate the microorganism in situ, bypassing the senescent immune system's difficulty in recruiting more leukocyte cells.^[^ ^59^ ^]^ The critical point is that exacerbated inflammation would not be helpful in controlling the microorganisms in older individuals.^[^ ^60^ ^]^

It is important to highlight that after light stimulation, the PS enter an excited state and transfer their energy to molecular oxygen or nitrogen present in the cellular environment. This energy transfer leads to the formation of reactive oxygen species (ROS), including hydroxyl radicals, hydrogen peroxide, and singlet oxygen.^[^ ^33^ ^]^ Due to their highly reactive nature, ROS have very short half‐lives and are rapidly degraded or neutralized in the cellular environment.^[^ ^61^ ^]^ The rapid degradation of these reactive species could explain the observed lack of differences between the *P. cincinnata* group without photoactivation and the control vehicle group. Furthermore, a limitation of our study is the relatively small number of animals used. Therefore, the ex vivo photoactivation in C57BL/6 animals might have influenced the results. Due to melanin's potential ability to interfere with PDT,^[^ ^27^, ^55^ ^]^ as previously stated, this led us to opt for an *ex vivo* activation. Nonetheless, in a study on resveratrol, Santos et al. observed that photoactivated resveratrol forms resveratrone, which could increase the duration of action of oxidative mechanisms and induce the clearance of MRSA, so in addition to ROS, a conformational change in the molecules due to light may be responsible for the bacterial clearance action.

Another interesting point of this study is that senescent animals produced the same cytokine levels as those in Vehicle, *P. cincinnata*, and aPDT groups. Despite this and the limitations of the small number of animals used in this study, cytokine behavior differed among groups. Other studies observed the differences in the production of the same cytokines analyzed here following photodynamic therapy. Still, they focused on different models, like young diabetic animals and young animals, both C57BL/6 lineages.^[^ ^24^, ^25^, ^26^, ^27^ ^]^ Furthermore, the aPDT group displayed a more distinct pattern of cytokine correlations, with a higher number of cytokines exhibiting negative correlations ‐ both among the pro‐inflammatory cytokines and with the anti‐inflammatory cytokine IL‐10 (Figure 6h). Senescence is accompanied by chronic and sterile low‐grade inflammation, in which the infection can exacerbate this process, causing dysregulation in the production of anti‐inflammatory cytokines, corroborating with more positive correlations among the analyzed cytokines,^[^ ^62^ ^]^ and consequently a greater imbalance in the inflammatory process. However, it appears that the use of *P. cincinnata* in PDT has overcome this.

Additionally, positive correlations were observed between IL‐10 and TNF‐α exclusively in the aPDT group, as well as between IL‐12p70 and IL‐17A in both the aPDT and *P. cincinnata* groups (Figure 6g,h), with these correlations becoming more pronounced in the aPDT group. It was recently demonstrated that the combined treatment of IL‐10+, IL‐12+, TNF‐α, decreased oxidative stress, CXCL8, and CXCR1 expression through the mitigation of the TNFR1‐IL‐1R‐NF‐κB inflammatory pathway in *S. aureus*.^[^ ^63^ ^]^ Furthermore, high levels of proinflammatory cytokines, particularly IL‐12, induce a subset of Th17 cells, which are IL‐17 producers and are important in neutrophilic recruitment, essential for the cutaneous defense of *S. aureus*.^[^ ^64^, ^65^, ^66^ ^]^ Although cytokines are produced at the same level between groups, their interaction differs depending on the context/treatment. Recent studies demonstrate that these analyses are not limited to diagnosing a single biomarker, as they use different inflammatory biomarkers and establish a network of interaction between them, demonstrating the importance of expanded analysis since our body depends on the action of the interaction of other systems.^[^ ^62^, ^67^ ^]^

## 4 Conclusion

This work is of great importance in studying new candidates for photosensitizers, which may be appropriate to control infections caused by antibiotic‐resistant bacteria. Here, we demonstrated the photodynamic activity of different species of the genus *Passiflora* tested for the first time, and one of them was highly effective in a senescence model of intradermal infection with MRSA. This research lays the groundwork for developing targeted aPDT strategies using *Passiflora* extracts and emphasizes the need to explore new photosensitizers derived from under‐researched plants. Our results are promising, primarily because little is known about how aPDT acts in a model of senescence using black mice and bring to light *P. cincinnata* as an important photosensitizer.

## 5 Experimental Section

### Collection and Preparation of the Plant Material

A population of *P. cincinnata* was identified and collected near the Southwest Bahia State University, Bem Querer Road, Km‐04 ‐ 3293, located in the municipality of Vitória da Conquista, Bahia (Latitude: 14° 53′ 3.975′' South, Longitude: 40° 47′ 59.137′' West). The *P. cincinnata* leaves were dried at 40 °C for 72 h in a circulating air oven. After drying, the leaves were crushed in a knife mill, yielding approximately 270 g of dry and crushed powder. Permission to access and study the genetic heritage (AE76E5D) was obtained through registration in the National System for the Management of Genetic Heritage and Associated Traditional Knowledge – SisGen/Brazil.

### *P. cincinnata* Extract Preparation and Partition

The dried and crushed leaves were subjected to maceration with 70% ethanol for 48 h, preceded by an ultrasound bath for 15 min at room temperature. Then, the material was filtered, the precipitate was resuspended in 70% ethanol and submitted to an ultrasound bath, and rested for 48 h. This procedure was repeated once more, and after 48 h, the extract was filtered and concentrated using a rotary evaporator. Part of the crude extract (10 grams) was suspended in 166.7 mL of hexane, homogenized in an ultrasonic bath, and filtered through a filtration system using qualitative filter paper, 80 grams and 11 cm in diameter (Qualy). The precipitate was resuspended in 166.7 mL of methanol: water (MeOH: H_2_O) (7:3 v/v) and partitioned with dichloromethane (CH_2_Cl_2_) (3 × 83 mL). 133.3 mL of H_2_O was added to the MeOH: H_2_O phase, and ethyl acetate (AcOEt) (3 × 83 mL) was partitioned. The 66.7 mL of H_2_O was added to the MeOH: H_2_O phase, and n‐butanol (BuOH) (4 × 83 mL) was partitioned. The BuOH phase was concentrated under reduced pressure, yielding the BuOH extract (1.7308 g = 17.3%). Part of the BuOH fraction (700 mg diluted in 3 mL of methanol at a time) was applied to a chromatographic column containing Sephadex LH – 20 (42 cm in height by 3.5 cm in diameter) and packed with methanol (MeOH). This sample was eluted with MeOH, maintaining a flow rate of ≈1.5 mL min^−1^. After eluting the dead volume (90 mL), a subfraction of ≈60 mL was collected, concentrated in a rotary evaporator, lyophilized, and stored in the refrigerator (4 °C).

### *P. edulis* and *P. alata* Extract

The *P. edulis* extract was obtained from the supplier Organic Compoundi LTDA (CNPJ: 18.186.547/0001‐99), manufacturer batch: 21k25‐FL00‐00000. The *P. alata* extract from provider SM Empreendimentos Farmacêuticos LTDA (CNPJ: 44.015.477/0005‐40), manufacturer batch: 2 201 026. Both extracts are derived from the leaves of *Passiflora*.

### Scanning of *P. edulis*, *P. alata*, and *P. cincinnata* Extracts by UV–vis

To estimate at which wavelengths *P. edulis*, *P. alata*, and *P. cincinnata* extracts absorb light, a scanning analysis was performed using a molecular absorption spectrophotometer (UV‐1800 Shimadzu/UV Spectrophotometer), evaluating the interval between 200 and 800 nm. The *P. alata* and *P. edulis* extracts were scanned at 100 µg mL^−1^ concentration, while *P. cincinnata* in 10 µg mL^−1^, both diluted in distilled water, using the spectrum function.

### *Passiflora* Cytotoxicity Assay by MTT in HUVEC

The human umbilical vein endothelial cell line (HUVEC) was obtained from the American Type Culture Collection (ATCC). HUVECs were cultivated in RPMI 1640 medium supplemented with 10% fetal bovine serum, 2 mm L‐glutamine, 2 mm sodium pyruvate, 1 mm non‐essential amino acids, 100 U mL^−1^ penicillin, and 100 mg mL^−1^ of streptomycin at 37 °C in 5% CO_2_. HUVECs were seeded at 3 × 10^4^ per well in 96‐well microplates to assess cytotoxicity. After 24 h, a new medium containing *P. edulis* (2000, 1500, 1000, 500 or 100 µg mL^−1^), *P. alata* (2000, 1500, 1000, 500, and 100 µg mL^−1^), and *P. cincinnata* (500, 100, 50, 10, and 5 µg mL^−1^) or medium and diluent controls (distilled water) was added and incubated at 37 °C and 5% CO_2_ for 24 h. After treatment, cells were incubated with MTT (5 mg mL^−1^, 20 µL per well (MTT: 3‐(4,5‐dimethylthiazol‐2‐yl)‐2,5‐diphenyl tetrazolium bromide) for 3 h at 37 °C. Then, 100 µL per well of PBS containing 10% SDS and 0.01 m HCl (18 h, 37 °C, and 5% CO_2_) was added. The absorbance was read in a multiwell scanning spectrophotometer (Multiskan GO Microplate Spectrophotometer –Thermo Scientific) at 570 nm. The IC_50_ value, representing the *Passiflora* extracts concentrations that decrease viability to 50% (IC_50_), was calculated from the concentration‐response curve.

### Bacterial Load

The bacterial load was determined by spectrophotometry, as described by dos Santos et al. (2020; 2019; 2019b). The reference strain, methicillin‐resistant *Staphylococcus aureus* 43 300 (MRSA 43 300) was used in the experiment. At the time of culture, the samples were thawed at room temperature, plated on BHI Agar culture medium (Brain Heart Infusion, pH 7.4, HIMEDIA), and taken to the incubator (Prolab) for 18‐24 h at 37 °C.

### In Vitro aPDT Experimental Design

*P. edulis* and *P. alata* were diluted in distilled water and used in concentrations of 2000, 1500, 1000, 500, and 100 µg mL^−1^. At the same time, they were tested at concentrations of 100, 50, and 10 µg mL^−1^ for *P. cincinnata*. These concentrations were defined after screening a wide range of concentrations for each species of *Passiflora*. To evaluate the *Passiflora* extracts' photodynamic effect, 10 µL, containing 1‐5 × 10^6^ CFU (Colony‐forming units) of MRSA was added to the 24 wells of the culture plates. Without blue LED light, the bacteria were solubilized in 990 µL of saline in the control group. In the group containing *Passiflora* extracts, 10 µL of MRSA + 10 µL of extract (at the specified concentrations) + 980 µL of sterile saline were placed in each well. Light control groups also received 10 µL of MRSA + 990 µL of saline + incidence of blue LED light (450 ± 20 nm) for 20 min and MRSA alone (without incidence of light and extract). Regarding the treatment groups, each group received 10 µL of MRSA + 10 µL of extract (at the indicated concentrations) + 980 µL of saline + exposure to blue LED light 20 min (56.4 J cm^−2^). After the addition of all components, a pre‐irradiation time of 5 min was respected in all groups. After that, 10 µL from each well was plated on BHI plates and incubated in an oven for 18–24 h at 37 °C. CFU quantification was performed using a colony counter (CP‐600 Plus).

### Animals

Sixteen C57BL/6 mice, ≈2 years old, were used in this experiment. The use of the C57BL/6 mouse lineage in aPDT is justified by its high melanin concentration, an aspect often overlooked in aPDT investigations. This choice aims to address the limited number of previous aPDT studies conducted in this animal lineage. However, despite this gap, some studies have already standardized the intradermal infection model for MRSA and aPDT in C57BL/6 mice, providing the methodological basis for the development of this work.^[^ ^25^, ^27^ ^]^ The animals were housed in the vivarium of the Federal University of Bahia ‐ Multidisciplinary Institute in Health ‐ *Campus* Anísio Teixeira, in an environment at a temperature of 25 °C, with a photoperiod of 12h:12 h light/dark, with food and water provided ad libitum. The Committee for Ethics in the Use of Animals (CEUA) IMS‐CAT UFBA approved all animal use procedures under protocol number 113/2022.

### In Vivo aPDT Experimental Design

The animals were divided into three groups (*n* = 5–6 per group), with both ears inoculated with MRSA, as follows: 1) Control animals (*n* = 6) infected with 10^8^ CFU of MRSA in 10 µL; 2) Control animals (*n* = 5) infected with 10^8^ CFU of MRSA with butanolic fraction of *P. cincinnata* (without photoactivation); 3) Animals (*n* = 5) infected with 10^8^ CFU of MRSA subjected to PDT treatment photosensitizing butanolic fraction of *P. cincinnata* at a concentration of 100 µg in 10 µL (concentration defined based on in vitro tests). All procedures were performed with animals anesthetized using xylazine and ketamine at 10 and 50 mg k^−1^g^−1^ doses. After 24 h of infection, aPDT animals were treated with butanolic fraction *P. cincinnata* intradermally (10 µL containing 100 µg) in both ears, previously photoactivated (ex vivo) with blue LED light (450 nm) for 180 s (13.5 J cm^−2^), with the equipment positioned 1 cm from the microtube, according to the protocol for *ex vivo* activation developed by Muniz et al. (2021). The animals in the MRSA control group were exposed to the stimulus with 10 µL of the vehicle. On the other hand, the PS control animals were treated with the butanolic fraction *P. cincinnata* (100 µg in 10 µL) without light activation. The entire procedure was performed with the animals anesthetized using xylazine and ketamine at 10 and 50 mg k^−1^g^−1^. After 72 h of infection, the animals were euthanized for the collection of lymph nodes and ears. The euthanasia of the animals was performed by deepening anesthesia with xylazine and ketamine at doses of 100 and 500 mg k^−1^g^−1^, respectively.

### Weighing of the Animals

The animals were weighed on an analytical balance during all experiment days at the same hour. The weights were recorded, and the percentage of weight loss was calculated based on the initial weight of each animal.

### Ear Bacterial Load

The right ears were collected and macerated in 1 mL of sterile saline, and then plated using 50 µL in 20 mL of BHI medium with oxacillin (6 µg mL^−1^). The plates were incubated in an oven for 18–24 h at 37 °C. CFU quantification was performed using a colony counter (CP‐600 Plus).

### Histopathology

The left ears of the animals were collected after pre‐defined times at the euthanasia points of the animals and were immediately fixed in methacarn (70% methanol, 20% chloroform, and 10% glacial acetic acid). After processing and hydration, the ears were embedded in paraffin and sectioned (4 µm) in a microtome. Sections were stained with hematoxylin and eosin. The stained sections were visualized under optical microscopy (Nikon Eclipse Ei), and the morphometric analysis was performed on photomicrographs of 20 fields per slide, obtained with the PrimeCam Intervision software (Prime Life Science), using a 40X objective.

### Cytokine Concentration

Retromaxillary lymph nodes from the mice were collected for cytokine concentration. Lymph nodes were macerated in 1 mL of sterile saline and centrifuged (1500 rpm at 4 °C for 10 min). The supernatant was collected and stored at a temperature of −80 °C for cytokine measurement (TNF‐α, IL‐1β, IL‐12p70, IL‐17A, IL‐10) quantified according to the manufacturer's instructions (Invitrogen ‐ ThermoFisher).

### Statistical Analyses

Statistical analyses were performed using the GraphPad‐Prism version 9.1.0 program (GraphPad Software). The Shapiro‐Wilk test was used to assess normality. Based on the data distribution, multiple comparisons were performed through the appropriate test (parametric ‐ Ordinary One‐way ANOVA or non‐parametric ‐ Kruskal‐Wallis), with its referred post‐test (in which different groups will be evaluated and compared to each other), Tukey or Dunn. Numerical variables were expressed as the mean ± standard error. Statistical differences were considered significant when *p* <0.05, using a 95% confidence interval. Multivariate analyses were performed using RStudio version 1.2.5001© 2009–2019 RStudio, Inc. Corrplot version 0.84 was used for correlation, and p (*p*) values were indicated based on Spearman or Pearson rank correlation tests (*p* < 0.05; *p* < 0.01; *p* < 0.001).

### Ethics Approval

The Committee for Ethics in the Use of Animals (CEUA) IMS‐CAT UFBA approved all animal use procedures under protocol number 113/2022.

## Conflict of Interest

The authors declare no conflict of interest.

## Author Contributions

C.V.G. performed conceptualization, validation, formal analysis, investigation, writing ‐ original draft, and writing ‐ review & editing. M.P.L.G. performed validation, methodology, and investigation. P.H.B.L., I.P.R.M., and I.S.R. performed validation, methodology, and investigation. M.E.S.d.O., C.O.L.d.M., S.L.d.O., and M.E.S.F. performed validation and methodology. C.S.G., L.C.S., and P.M.L. performed validation, methodology, and resources. D.S.L. performed conceptualization, methodology, formal analysis, and data curation. J.G.A. performed conceptualization, methodology, resources, and supervision. R.A.A.d.S. performed term, conceptualization, data curation, methodology, resources, supervision, project administration, and funding acquisition.

## Data Availability

The data that support the findings of this study are available from the corresponding author upon reasonable request.

## References

[1] M. Falcone , G. Tiseo , Curr. Opin. Infect. Dis. 2023, 36, 102. 36718942 10.1097/QCO.0000000000000907 PMC10325572

[2] M. Venditti , M. Falcone , A. Micozzi , P. Carfagna , F. Taglietti , P. F. Serra , P. Martino , Infectious Disorders 2003, 88, 923. 12935981

[3] L. Neloska , K. Damevska , A. Nikolchev , L. Pavleska , B. Petreska‐Zovic , M. Kostov , Open Access Maced J Med Sci 2023, 4, 423. 10.3889/oamjms.2016.094 PMC5042627 27703567

[4] H. Yousef , M. Alhajj , A. O. Fakoya , S. Sharma , In: Anatomy, Skin (Integument) Epidermis 2024, StatPearls [Internet]. Treasure Island (FL) StatPearls Publishing, 2025, https://www.ncbi.nlm.nih.gov/books/NBK470464/.

[5] E. Russell‐Goldman , G. F. Murphy , Am J Pathol 2020, 190, 1356. 32246919 10.1016/j.ajpath.2020.03.007 PMC7481755

[6] G. Y. C. Cheung , J. S. Bae , M. Otto , Virulence 2023, 12, 547. 10.1080/21505594.2021.1878688 PMC7872022 33522395

[7] N. A. Turner , B. K. Sharma‐Kuinkel , S. A. Maskarinec , E. M. Eichenberger , P. P. Shah , M. Carugati , T. L. Holland , V. G. Fowler , Nat. Rev. Microbiol. 2023, 17, 203. 10.1038/s41579-018-0147-4 PMC6939889 30737488

[8] P. Nandhini , P. Kumar , S. Mickymaray , A. S. Alothaim , J. Somasundaram , M. Rajan , Antibiotics 2022, 11, 606. 35625250 10.3390/antibiotics11050606 PMC9137690

[9] A. H. Hasanpour , M. Sepidarkish , A. Mollalo , A. Ardekani , M. Almukhtar , A. Mechaal , S. R. Hosseini , M. Bayani , M. Javanian , A. Rostami , Antimicrob Resist Infect Control 2023, 12, 4. 36709300 10.1186/s13756-023-01210-6 PMC9884412

[10] A. Bleve , F. Motta , B. Durante , C. Pandolfo , C. Selmi , S. A. Immunosenescence , Clin. Rev. Allergy Immunol. 2023, 64, 123 35031957 10.1007/s12016-021-08909-7 PMC8760106

[11] World Health Organization , WHO Bacterial Priority Pathogens List, https://iris.who.int/bitstream/handle/10665/376776/9789240093461‐eng.pdf?sequence=1, Jan 9 2025.

[12] T. M. Uddin , A. J. Chakraborty , A. Khusro , B. R. M. Zidan , S. Mitra , T. B. Emran , K. Dhama , Md. K. H. Ripon , M. Gajdács , M. U. K. Sahibzada , Md. J. Hossain , N. Koirala , J Infect Public Health 2021, 14, 1750. 34756812 10.1016/j.jiph.2021.10.020

[13] G. Mancuso , A. Midiri , E. Gerace , C. Biondo , Pathogens 2023, 10, 1310. 10.3390/pathogens10101310 PMC8541462 34684258

[14] R. Selvarajan , C. Obize , T. Sibanda , A. L. K. Abia , H. Long , Antibiotics 2022, 12, 28. 36671228 10.3390/antibiotics12010028 PMC9855083

[15] M. I. Hutchings , A. W. Truman , B. Wilkinson , Curr. Opin. Microbiol. 2019, 51, 72. 31733401 10.1016/j.mib.2019.10.008

[16] M. Vestergaard , D. Frees , H. Ingmer , Microbiol Spectr 2019, 7. 10.1128/microbiolspec.gpp3-0057-2018 PMC11590431 30900543

[17] M. Younis , T. A. Tabish , C. Firdharini , M. Aslam , M. Khair , D. H. Anjum , X. Yan , M. Abbas , ACS Appl. Mater. Interfaces 2025. 10.1021/acsami.5c03250 PMC12086768 40301105

[18] M. Tasleem , A. M. Matouk , M. Abbas , ChemBioChem 2025, 26, 202500122. 10.1002/cbic.202500122 40183352

[19] N. Saeed , A. Atiq , F. Rafiq , I. Khan , M. Atiq , M. Saleem , D. H. Anjum , Z. Usman , M. Abbas , Sci. Rep. 2024, 14, 26398. 39488657 10.1038/s41598-024-78320-7 PMC11531511

[20] H. K. Sharma , P. Gupta , D. Nagpal , M. Mukherjee , V. S. Parmar , Fitoterapia 2023, 168, 105554. 37270161 10.1016/j.fitote.2023.105554

[21] M. O. Falade , P. M. Duffin , M. O. Falade , Natural Compounds Targeting ABC Transporters and MecA to Combat MRSA Antibiotic Resistance , Jun 2025, https://www.preprints.org/manuscript/202501.1771/v1.

[22] M. Kashi , M. Noei , Z. Chegini , A. Shariati , Front. Pharmacol. 2024, 15, 1491363. 39635434 10.3389/fphar.2024.1491363 PMC11615405

[23] T. W. Wong , E. C. Wu , W. C. Ko , C. C. Lee , L. I. Hor , I. H. Huang , Dermatologica Sinica 2018, 36, 8.

[24] D. P. dos Santos , D. P. S. Lopes , S. P. de Melo Calado , C. V. Gonçalves , I. P. R. Muniz , I. S. Ribeiro , M. P. L. Galantini , R. A. A. da Silva , J Photochem Photobiol B 2019, 191, 107. 30599381 10.1016/j.jphotobiol.2018.12.011

[25] I. P. R. Muniz , M. P. L. Galantini , I. S. Ribeiro , C. V. Gonçalves , D. P. dos Santos , T. C. Moura , E. S. Silva , N. R. Silva , B. P. Cipriano , T. M. L. Correia , T. de Jesus Soares , L. M. de Freitas , D. J. Costa , R. A. A. da Silva , J Photochem Photobiol B 2021, 224, 112325. 34598018 10.1016/j.jphotobiol.2021.112325

[26] D. P. dos Santos , M. P. L. Galantini , I. S. Ribeiro , I. P. R. Muniz , I. S. Pereira , R. A. A. da Silva , Lasers Med Sci 2020, 35, 1341. 31900691 10.1007/s10103-019-02942-x

[27] D. P. dos Santos , D. P. Soares Lopes , R. C. de Moraes , C. Vieira Gonçalves , L. Pereira Rosa , F. C. da Silva Rosa , R. A. da Silva , Photodiagnosis Photodyn Ther 2019, 25, 227. 30630110 10.1016/j.pdpdt.2019.01.005

[28] P. P. Almeida , Í. S. Pereira , K. B. Rodrigues , L. S. Leal , A. S. Marques , L. P. Rosa , F. C. da Silva , R. A. A. da Silva , Lasers Med Sci 2017, 32, 1337. 28646389 10.1007/s10103-017-2247-1

[29] C. W. Tseng , P. A. Kyme , A. Arruda , V. K. Ramanujan , W. Tawackoli , G. Y. Liu , PLoS One 2012, 7, 41454. 10.1371/journal.pone.0041454 PMC3406035 22844481

[30] I. Waldmann , T. Schmid , J. Prinz , B. Mühleisen , R. Zbinden , L. Imhof , Y. Achermann , Photodiagnosis Photodyn Ther 2020, 31, 101941. 32755635 10.1016/j.pdpdt.2020.101941

[31] J. Huang , Q. Fan , L. Shi , J. Shen , H. Wang , Photodiagnosis Photodyn Ther 2024, 48, 104300. 39097252 10.1016/j.pdpdt.2024.104300

[32] A. S. C. Gonçalves , M. M. Leitão , J. R. Fernandes , M. J. Saavedra , C. Pereira , M. Simões , A. Borges , J Photochem Photobiol B 2024, 258, 112978. 39002192 10.1016/j.jphotobiol.2024.112978

[33] J. H. Correia , J. A. Rodrigues , S. Pimenta , T. Dong , Z. Yang , Pharmaceutics 2021, 13, 1332. 34575408 10.3390/pharmaceutics13091332 PMC8470722

[34] R. R. Allison , G. H. Downie , R. Cuenca , X. H. Hu , C. J. H. Childs , C. H. Sibata , Photodiagnosis Photodyn Ther 2004, 1, 27. 25048062 10.1016/S1572-1000(04)00007-9

[35] H. Abrahamse , M. R. Hamblin , Biochem. J. 2016, 473, 347. 26862179 10.1042/BJ20150942 PMC4811612

[36] E. Polat , K. Kang , Biomedicines 2021, 9, 584. 34063973 10.3390/biomedicines9060584 PMC8224061

[37] M. J. Garland , C. M. Cassidy , D. Woolfson , R. F. Donnelly , Future Med Chem. 2009, 1, 667. 21426032 10.4155/fmc.09.55

[38] G. M. Costa , A. C. Gazola , S. M. Zucolotto , L. Castellanos , F. A. Ramos , F. H. Reginatto , E. P. Schenkel , Revista Brasileira de Farmacognosia 2016, 26, 451.

[39] I. L. Gadioli , S. B. M. da Cunha , M. V. O. de Carvalho , A. M. Costa , L. Pineli , L. L. O. de Pineli , Crit Rev Food Sci Nutr. 2017, 58, 785. 27645583 10.1080/10408398.2016.1224805

[40] T. Barbosa Santos , F. P. de Araujo , A. F. Neto , S. T. de Freitas , J. de Souza Araújo , O. de , S. B. Vilar , A. J. Brito Araújo , M. S. Lima , International Journal of Fruit Science 2021, 21, 255.

[41] A. E. B. P. Leal , A. P. de Oliveira , R. F. D. Santos , J. M. D. Soares , E. M. D. Lavor , M. C. Pontes , J. T. D. Lima , A. D. D. Santos , J. C. Tomaz , G. G. D. Oliveira , F. C. Neto , N. P. Lopes , L. A. Rolim , J. R. G. D. Almeida , Nat. Prod. Res. 2020, 34, 995. 30584781 10.1080/14786419.2018.1548445

[42] É. M. de Lavor , A. E. B. P. Leal , A. W. C. Fernandes , F. P. R. D. A. Ribeiro , J. M. De Barbosa , M. Gama e Silva , R. B. D. A. Teles , L. F. D. S. Oliveira , J. C. Silva , L. A. Rolim , I. R. A. de Menezes , J. R. G. D. S. Almeida , Phytomedicine 2018, 47, 58. 30166109 10.1016/j.phymed.2018.04.052

[43] I. S. Ribeiro , I. P. R. Muniz , M. P. L. Galantini , C. V. Gonçalves , P. H. B. Lima , E. S. Silva , N. R. Silva , F. C. Rosa , L. P. Rosa , D. J. Costa , J. G. Amaral , Photochem. Photobiol. Sci. 2023, 22, 2877. 37923909 10.1007/s43630-023-00495-1

[44] I. S. Ribeiro , I. P. R. Muniz , M. P. L. Galantini , C. V. Gonçalves , P. H. B. Lima , N. R. Silva , S. L. de Oliveira , M. S. Nunes , A. K. Novaes , M. E. de Oliveira , D. J. Costa , Photochem. Photobiol. Sci. 2024, 23, 561. 38372844 10.1007/s43630-024-00539-0

[45] L. R. da Fonseca , R. A. Rodrigues , A. S. Ramos , J. D. da Cruz , J. L. P. Ferreira , J. R. A. Silva , A. C. F. Amaral , Scientific World Journal 2020.

[46] X. He , F. Luan , Y. Yang , Z. Wang , Z. Zhao , J. Fang , M. Wang , M. Zuo , Y. Li , Front Pharmacol 2020, 11, 617. 32508631 10.3389/fphar.2020.00617 PMC7251050

[47] A. E. B. Pereira Leal , É. M. de Lavor , J. de Menezes Barbosa , M. T. de Moura Fontes Araújo , C. dos Santos Cerqueira Alves , R. G. de Oliveira Júnior , Á. A. N. de Lima , J. R. G. da Silva Almeida , Curr. Top. Med. Chem. 2022, 22, 2315. 35986522 10.2174/1568026622666220819160923

[48] Y. W. Ni , K. H. Lin , K. H. Chen , C. W. Wu , C. Y. Sen , Plants 2020, 9, 633.32429275

[49] D. Da Silva Francischini , A. P. Lopes , M. L. Segatto , A. M. Stahl , V. G. Zuin , BMC Chem 2020, 14, 1. 32968737 10.1186/s13065-020-00710-5 PMC7501698

[50] A. J. Vargas , D. S. Geremias , G. Provensi , P. E. Fornari , F. H. Reginatto , G. Gosmann , E. P. Schenkel , T. S. Fröde , Fitoterapia 2007, 78, 112. 17215089 10.1016/j.fitote.2006.09.030

[51] L. E. M. Brandaõ , D. A. M. F. Nôga , A. L. Dierschnabel , C. L. D. C. Campêlo , Y. D. S. R. Meurer , R. H. Lima , R. C. G. J. Engelberth , J. S. Cavalcante , C. A. Lima , M. Marchioro , C. D. S. Estevam , J. R. Santos , J. R. Silva , A. M. Ribeiro , Evid Based Complement Alternat Med 2017. 10.1155/2017/8429290 PMC5556616 28835767

[52] A. L. A. Siebra , L. R. Oliveira , A. O. B. P. B. Martins , D. C. Siebra , R. S. Albuquerque , I. C. S. Lemos , G. A. Delmondes , S. R. Tintino , F. G. Figueredo , J. G. M. da Costa , H. D. M. Coutinho , I. R. A. Menezes , C. F. B. Felipe , M. R. Kerntopf , Saudi J Biol Sci 2018, 25, 37. 29379354 10.1016/j.sjbs.2016.01.019 PMC5775088

[53] A. A. L. de Siebra , I. C. S. Lemos , A. G. de Delmondes , L. R. de Oliveira , A. O. B. P. B. Martins , C. D. de Siebra , H. D. M. Coutinho , R. S. Albuquerque , N. F. Leite , J. G. M. da Costa , I. R. A, de Menezes , M. R. Kerntopf , Revista Cubana de Plantas Medicinales 2014, 19, 319.

[54] S. C. Guimarães , M. P. Lima , C. B. M. Sousa , C. C. Araújo , R. S. V. Carmo , G. L. Borges , D. B. Silva , F. M. Leite , A. B. G. Damasceno , J. G. Amaral , ACS Sustain Chem Eng 2025, 13, 3907.

[55] C. M. Cassidy , R. F. Donnelly , M. M. Tunney , J Photochem Photobiol B 2010, 99, 62. 20207552 10.1016/j.jphotobiol.2010.02.004

[56] A. C. Shaw , D. R. Goldstein , R. R. Montgomery , Nat. Rev. Immunol. 2013, 13, 12. 10.1038/nri3547 PMC4096436 24157572

[57] H. Lee , Y. Hong , M. Kim , Int. J. Mol. Sci. 2021, 22, 12489. 34830368 10.3390/ijms222212489 PMC8624050

[58] E. M. Crimmins , C. E. Finch , Proc Natl Acad Sci U S A 2005, 103, 498. 16387863 10.1073/pnas.0501470103 PMC1326149

[59] M. De Martinis , M. Modesti , L. Ginaldi , Immunol. Cell Biol. 2004, 82, 415. 15283852 10.1111/j.0818-9641.2004.01242.x

[60] C. Shive , P. Pandiyan , Frontiers in Aging 2022, 3, 840827. 35821823 10.3389/fragi.2022.840827 PMC9261323

[61] K. Jomova , S. Y. Alomar , S. H. Alwasel , E. Nepovimova , K. Kuca , M. Valko , Arch. Toxicol. 2024, 98, 1323. 38483584 10.1007/s00204-024-03696-4 PMC11303474

[62] C. V. Gonçalves , I. S. Ribeiro , M. P. L. Galantini , I. P. R. Muniz , P. H. B. Lima , G. S. Santos , R. A. A. da Silva , Exp. Gerontol. 2022, 170, 112005. 36341786 10.1016/j.exger.2022.112005

[63] P. Dutta , B. Bishayi , Int. Immunopharmacol. 2023, 120, 110297. 37207443 10.1016/j.intimp.2023.110297

[64] J. S. Cho , E. M. Pietras , N. C. Garcia , R. I. Ramos , D. M. Farzam , H. R. Monroe , J. E. Magorien , A. Blauvelt , J. K. Kolls , A. L. Cheung , G. Cheng , R. L. Modlin , L. S. Miller , J. Clin. Invest. 2010, 120, 1762. 20364087 10.1172/JCI40891 PMC2860944

[65] E. Volpe , N. Servant , R. Zollinger , S. I. Bogiatzi , P. Hupé , E. Barillot , V. Soumelis , Nat. Immunol. 2008, 9, 650. 18454150 10.1038/ni.1613

[66] A. Belpaire , N. van Geel , R. Speeckaert , Front Immunol 2022, 13, 932265. 35967358 10.3389/fimmu.2022.932265 PMC9367984

[67] M. P. L. Galantini , I. S. Ribeiro , C. V. Gonçalves , I. P. R. Muniz , P. H. B. Lima , G. S. Santos , R. A. da Silva , Exp. Gerontol. 2022, 167, 111905. 35918042 10.1016/j.exger.2022.111905

